# Fatigue and Vitamin D Status in Iranian Female Nurses

**DOI:** 10.5539/gjhs.v8n6p196

**Published:** 2015-11-17

**Authors:** Negin Masoudi Alavi, Mahla Madani, Zohre Sadat, Hamed Haddad Kashani, Mohammad Reza Sharif

**Affiliations:** 1 Trauma Nursing Research Center, Kashan University of Medical Sciences, Kashan, Iran; 2 Student Research Committee, Kashan University of Medical Sciences, Kashan, Iran; 3 Anatomical Sciences Research Center, Kashan University of Medical Sciences, Kashan, Iran; 4 Department of Pediatrics, Kashan University of Medical Sciences, Kashan, Iran

**Keywords:** fatigue, Vitamin D deficiency, female, nurses, Iran

## Abstract

**Introduction:**

Given that nurses are among professions with frequent problems of fatigue, and given the nature of their profession that provides little exposure to sunlight and the subsequent deficiency of vitamin D, the present study examined the relation between fatigue and circulating vitamin D levels in female nurses working in Shahid Beheshti Hospital, Kashan, Iran in 2013.

**Material and Methods:**

This cross-sectional study was conducted in 200 female nurses working in Shahid Beheshti Hospital. To measure fatigue, fatigue questionnaire containing 9 questions eliciting the subject’s feeling in scales of 1 to 7, getting a possible score of 9 to 63, and Visual Analogue Scale in which nurses specified their fatigue in a band of zero to 10 were used. The 25-hydroxyvitamin D, which is the most important vitamin D metabolite, also was determined. The data was analyzed by SPSS-16. The Pearson’s correlation of coefficients, t-test, and multiple regression analysis were used in this study.

**Results:**

The mean fatigue score of nurses was 38.76±12.66 in questionnaire and 5.73±2.12 in Visual Analog Scale. The 89 per cent of nurses suffered from vitamin D deficiency, 9.5 percent of them had normal level and 1.5 per cent had toxicity level of vitamin D. There was a significant relationship between vitamin D level and fatigue scores (P<0.0001), and visual fatigue scores (P<0.0001). According to multivariate regression analysis, vitamin D level accounted for 13 per cent of the fatigue based on data on questionnaire and 18.6 per cent of fatigue according to Visual Analog Scale.

**Conclusion:**

High prevalence of fatigue among nurses could be attributed to vitamin D deficiency.

## 1. Introduction

Nursing is among the occupations fraught with tension and fatigue (Askari et al., 2007). Working in three shifts (Øyane et al., 2013; Eldevik et al., 2013), in difficult settings such as oncology or emergency wards (Flarity et al., 2013; Potter et al., 2010), and caring of incurable patients put a considerable psychological, spiritual, and physical pressures on nurses (AfkhamEbrahimi et al., 2004). As a result, fatigue is a common feeling among nurses (Askari et al., 2007). In a study 43.4% of nurses reported excessive fatigue (Eldevik et al., 2013). Raftopolous and colleagues also reported that 91.9% of Cypriot nurses had fatigue (Raftopoulos et al., 2004). The prevalence of fatigue in adults has been 27% which is less than its prevalence in nurses (Bates et al., 1993). The prevalence of Chronic Fatigue Syndrome (CFS), considered as being the extreme case of the fatigue, has been 0.3 to 1% in public while its prevalence is almost twice, among special care nurses (Pawlikowska et al., 1994). A research indicated that almost 50 percent of those contacted the institute handling CFS patients were nurses (Pawlikowska et al., 1994). These nurses gradually felt that they no longer could work with the patients and felt difficulty providing caring duties. This factor diminishes their self-confidence and cause feeling of failure (Abdi 2008). Nurses, who feel fatigue, could not be good caregivers to the patients (Kuerer et al., 2007). Occupational fatigue cause higher probability of errors, and decreased quality of care giving services (Rassouli et al., 2011). This would extremely impact the health care provision quality (Nasri 2004). Micronutrients have extensive impact on the body (Kennedy et al., 2010). Vitamin D as a micronutrient is accessible in enriched nutritionals and supplements as ergocalciferol-D2 and cholecalciferol-D3. In addition to confirmed role in bone growth and health, and preventing esteomalacia, it has important roles in cell differentiation, multiplication and growth in muscles, pancreas and parathyroid (Mahan et al., 2012; Targher et al., 2006). The vitamin D prevents cancer cell multiplication and increases tumor controlling activity (Tse et al 2007). It also effects on thyroid (Qu et al., 2012); muscular-nervous system (Salacinski et al., 2013); immune (Fragoso et al., 2012), and autoimmune system activity (Askmark et al., 2012). A lower level of circulating vitamin D is possibly inversely associated with some cancers, Type-2 diabetes, metabolic syndrome, and cardio-vascular diseases (Targher et al., 2006). An average human receives vitamin D with adequate amount of exposure to sunlight and foods containing vitamin D. However, increasing evidence suggests that the received vitamin D is deficient. Insufficient vitamin D is prevalent globally regardless of age and health conditions (Mahan et al., 2012; Hoeck et al., 2011). The women, who cover their body, suffer more severe vitamin D deficiency (Ojah et al., 2012). Lower levels of vitamin D can cause fatigue (Pérez 2007). Some studies have found that intake of vitamin D supplement could wield positive effects on fatigue (Askmark et al., 2012; Hoeck et al., 2011). A study on patients diagnosed with multiple myeloma found that vitamin D deficiency caused pain, and fatigue (Kimlin et al. 2007). In another study, 65 percent of patients diagnosed with chronic fatigue syndrome after head injuries, were deficient in vitamin D (Schnieders et al., 2012). A study in the Netherlands, however, found no relationship between level of circulating vitamin D and fatigue in patients with multiple sclerosis (Knippenberg et al., 2011). The majority of studies on the association of vitamin D and fatigue produced diverse results and these studies are mostly on patients with different chronic conditions. The relationship between vitamin D and fatigue in healthy people has not been investigated sufficiently. Given that nurses are among professions with frequent problems of fatigue, and given the nature of their profession that provides little exposure to sunlight and the subsequent deficiency of vitamin D, the present study examined the relation between fatigue and circulating vitamin D levels in female nurses working in Shahid Beheshti Hospital, Kashan in 2013.

## 2. Materials and Methods

This cross-sectional study was conducted in the general hospital of Shahid Beheshti in Kashan, Iran, in August 2013. Shahid Beheshti is a general hospital with 500 active beds and 526 full-time nurses. The 78 percent of nurses are female. Kashan is located in the center of Iran with sunny weather throughout the year.

The sample population included female nurses working in Shahid Beheshti Hospital in emergency, surgery, and, internal wards. First, names of all female nurses were listed and numbered. Then, the participant nurses were randomly selected from the table of random numbers. If a nurse was reluctant to participate, and if she did not satisfy the study criteria, another nurse was selected randomly till 200 female nurses completed the study. The criteria of inclusion in the study were having no past record of chronic diseases such as diabetes, coronary heart diseases, thyroid disorders, depression, cancer, and muscular disorders; at least a year of employment in the hospital; and lack of supplementation of vitamin D during the previous month. The selected female nurses were interviewed, and after getting their written approval, they were asked to fill in the fatigue questionnaire. Then, their venous blood samples were taken in 6 mL syringes and were sent to Milad laboratory to determine vitamin D level. To measure fatigue, we used two instruments. First fatigue questionnaire containing 9 questions eliciting the subject’s feeling in scales of 1 to 7, getting a possible score of 9 to 63, with the greatest number indicating higher feeling of fatigue. The internal consistency of the questionnaire items has been determined to be 0.96 (Cronbach’s alpha coefficient) in Shahvarughi et al. (2010). The other instrument was Visual Analogue Scale in which nurses specified their fatigue in a band of zero to 10. To determine serum vitamin D level, we used its metabolite, since vitamin D is converted to 25-hydroxyvitamin D in liver, which is the most important vitamin D metabolite. The serum 25-hydroxyvitamin D level was determined through ELISA method and standard kit DIAsource after the coagulated venous blood was sent to laboratory. In line with ethics of the study, the informed consent was obtained from nurses and all participants were provided with results of their experiments, and those with vitamin D deficiency were informed. The data was analyzed by SPSS-16. To examine correlation between serum vitamin D and fatigue, we used Pearson’s correlation of coefficients, and after classifying vitamin D deficiency the chi-square test was used for data analysis. Taking fatigue level as a dependent variable, effective factors on it was examined by a multiple regression analysis.

## 3. Results

**Figure 1:**
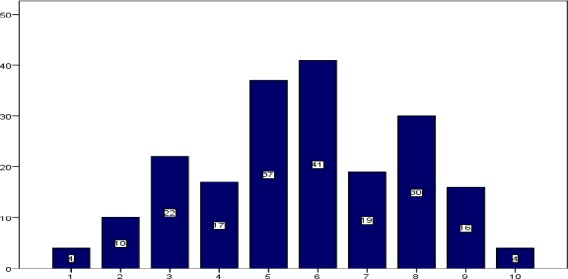
The number of nurses reported fatigue in visual analog scale

The mean age of the subjects was 32.05±5.22 years old. The 141 participant (70.5 percent) were married. The work experience was 9.19±4.86 years. Table 1 shows demographic information and mean levels of vitamin D. The mean fatigue score of nurses designated by the questionnaire was 38.76±12.66; mean scores of visual fatigue was 5.73±2.12. As given in Fig. 1, 4 participants (2 per cent) displayed no fatigue and 4 participants (2 per cent) displayed the maximum level of fatigue. 55 per cent of nurses reported fatigue score of higher than 5 in visual analog scale. Vitamin D levels were assigned to four groups of extreme deficiency (below 10 ng/ml), deficient (10-30 ng/ml), normal (30-100 ng/ml), and toxicity (above 100 ng/ml). Based on these categories, 91 participants (45.5 per cent) were extremely deficient in vitamin D; 87 participants (43.5 per cent) were deficient; 19 participants (9.5 per cent) had normal vitamin D level, and 3 participants (1.5 per cent) had toxicity level of vitamin D. We found a significant relationship between vitamin D level and number of shift work in a month (P<0.003); fatigue scores (P<0.0001), and visual fatigue scores (P<0.0001). However, we found no significant relationship between fatigue scores and hospital ward (P=0.07); age (P=0.4); marital status (P=0.7); number of children (P=0.3); work experience (P=0.5); numbers of shift work in a month (P=0.9); and the shift type (P=0.8).

**Table 1:** Demographic information and vitamin D levels

	Age	Years in job	Shift in month	Fatigue score	Visual fatigue score	Vitamin D levels
Mean	32.05	9.19	30.5	38.76	5.73	16.96
SD	5.22	4.86	3.37	12.66	2.12	21.12
Maximum	48	25	45	62	10	176.1
Minimum	22	1	20	10	1	3.5

Table 2 shows the correlation between vitamin D levels and variables of the study. According to multiple regression analysis, vitamin D level accounted for 13 per cent of the fatigue based on data on questionnaire (R2=0.133; adjusted R2=0.128). There was no significant relationship with other variables including age, work history, and the number of shift per month and fatigue.

**Table 2:** The relation between vitamin D levels and variables of the study

	Correlation with Vitamin D	Extreme deficiency	Deficiency	Normal	Dangerous	P-value
Age	R= -0.062 P=0.385	32.62±4.63	31.74±5.55	31±6.47	30.67±3.78	0.5
Work experience	R= -0.028 P=0.691	9.51±4.1	8.99±5.39	8.53±5.84	9.67±5.5	0.8
Number of shifts in month	R= 0.141 P=0.046	30.44±2.35	29.99±3.32	33.16±5.96	30.67±2.08	0.003
Fatigue scores	R= -0.364 P=0.0001	41. 9±12.3	39.06±11	25±11.62	22±1.73	0.001
Visual fatigue scores	R= -0.418 P=0.0001	6.38±2.27	5.68±1.49	3.37±1.77	2.33±0.57	0.001

Table 3 gives the regression analysis of the dependent variable fatigue according to data from the questionnaire and other variables.

**Table 3:** Regression analysis of the dependent variable fatigue according to data from the questionnaire and other variables

Variable	Beta	T	P-value
Vitamin D	-0.369	-5.416	0.001
Age	-0.016	-0.96	0.92
Work history	0.058	0.35	0.72
Number of shifts per month	0.063	0.9	0.36

The multiple regression analysis showed that vitamin D level accounted for 18.6 percent of fatigue according to Visual Analog Scale (R2=0.186; adjusted R2=0.161).

## 4. Discussion

We found that 89 per cent of nurses suffered from vitamin D deficiency. In terms of similar studies, a Thailand study found 95.4 percent of prevalence of vitamin D deficiency among nurses (Hattapornsawan et al., 2012). Another study in India, on 2119 health center workers in 18 different spots of country showed 94 percent deficiency and extreme deficiency of vitamin D (Beloyartseva et al., 2012). A related body of research in Qatar reported 95 percent vitamin D deficiency among female health care givers (Mahdy et al., 2010). In Iran a study by Shakiba carried out in hospital staff found that only 8.5 percent of the staff had a normal vitamin D levels, and the remaining showed different levels of deficiency. It seems that despite a sunny climate, the deficiency was however prevalent, with the sun playing no role in provision of vitamin D for the body (Shakiba et al., 2009). In Australia, Kimlin sought to respond to the question that whether a high radiation of UV in the environment helped to provide vitamin D needs of the body. The study found that 42.5 per cent of adults suffered moderate and severe deficiency of vitamin D, despite ample access to sunlight and sunny climate (Kimlin et al., 2007). This finding supports finding in Binkley’s research (Binkley et al., 2007). However, Chao investigated the issue in Canadian workers, and found the prevalence of vitamin D was 3 per cent (Chao et al., 2013). Thus it seems likely that there is a need for further research on sunlight and vitamin D production in the body, and other effective factors including nutritional habits and lifestyle (Vu et al., 2011). In present study, 55 per cent of nurses reported fatigue more than the average level. Ho’s study in Taiwan, who investigated work-induced fatigue among health workers, indicated that 37.1 per cent of nurses suffered fatigue, which was rather high compared to other health workers. Among the reasons cited as the probable causes were long working hours, changes in work shift, and the subsequent insomnia. Ho’s work reported that nurses in Taiwan worked more than 8 years in service, cited as a cause of fatigue (Ho et al., 2013). Another study in Colombia on 745 nurses found a positive and significant correlation between hectic work shifts and the shift numbers and fatigue. The work place atmosphere contributed to the fatigue as well (Barker et al., 2011). In Lombardo’s work, fatigue was attributed to different causes such as facing the death and suffering in patients (Lombardo et al., 2011). Due to nature of their job, nurses are more exposed to fatigue than other working groups (Sammartino et al., 2012). In the present study, a vitamin D level was associated with fatigue. In German context, Merlo reported fatigue in individuals with vitamin D deficiency (Merlo et al., 2012). However, Brunner carried out a direct interventional research to investigate the effect of vitamin D and calcium on physical performance of healthy women. Calcium and vitamin D intake had no effect on physical performance compared to placebo injection (Brunner et al., 2008). Vitamin D has had a greater impact on life different aspects in addition to inducing fatigue. A study carried out in Turkey on healthy premenopausal women to investigate circulating vitamin D level and quality of life found that women with vitamin D deficiency had a lower quality of life (Ecemis et al., 2013). Vitamin D supplements are highly recommended to alleviate the effect of the deficiency in target populations (McAlindon et al., 2013).

## 5. Conclusion

High prevalence of fatigue among nurses could be attributed to vitamin D deficiency. Thus, it is highly recommended that an interventional study be carried out to examine the impact of vitamin D on fatigue among the nurses.
